# Preexposure and Postexposure Prophylaxis of Rabies With Adeno-Associated Virus Expressing Virus-Neutralizing Antibody in Rodent Models

**DOI:** 10.3389/fmicb.2021.702273

**Published:** 2021-08-19

**Authors:** Fei Huang, Meishen Ren, Jie Pei, Hong Mei, Baokun Sui, Qiong Wu, Benjie Chai, Ruicheng Yang, Ming Zhou, Zhen F. Fu, Huiping Zhou, Ling Zhao

**Affiliations:** ^1^State Key Laboratory of Agricultural Microbiology, Huazhong Agricultural University, Wuhan, China; ^2^Key Laboratory of Preventive Veterinary Medicine in Hubei Province, College of Veterinary Medicine, Huazhong Agricultural University, Wuhan, China; ^3^School of Basic Medicine, Hubei University of Science and Technology, Xianning, China

**Keywords:** rabies viruses, adeno-associated virus, virus-neutralizing antibody, preexposure prophylaxis, postexposure prophylaxis, rodent models

## Abstract

Rabies, a fatal disease in humans and other mammals, is caused by the rabies virus (RABV), and it poses a public health threat in many parts of the world. Once symptoms of rabies appear, the mortality is near 100%. There is currently no effective treatment for rabies. In our study, two human-derived RABV-neutralizing antibodies (RVNA), CR57 and CR4098, were cloned into adeno-associated virus (AAV) vectors, and recombinant AAVs expressing RVNA were evaluated for postexposure prophylaxis after intrathecal injection into RABV-infected rats. At 4days post-infection with a lethal dose of RABV, 60% of the rats that received an intrathecal injection of AAV-CR57 survived, while 100% of the rats inoculated with AAV-enhanced green fluorescent protein (EGFP) succumbed to rabies. Overall, these results demonstrate that AAV-encoding RVNA can be utilized as a potential human rabies postexposure prophylaxis.

## Introduction

Rabies is a fatal disease caused by the rabies virus (RABV), and it is estimated that rabies causes 59,000 deaths annually, mostly in developing countries (Jackson, 2003; Fooks et al., 2014). RABV is a neurotropic virus of the genus Lyssavirus in the family *Rhabdoviridae*, with a single-strand negative-sense RNA genome and five structural proteins (N, P, M, G, and L; Schnell et al., 2010). Rabies has a varied incubation time. Since its early symptoms are usually mild, rabies infection may not be recognized until it invades the central nervous system (CNS; Kelly and Strick, 2000). The replication of RABV may maintain at a low level in muscle cells (Charlton and Casey, 1981; Yamaoka et al., 2013) after infection by a bite from a rabid animal. It subsequently invades the peripheral nervous system (PNS) by retrograde axonal transport and replicates in the spinal cord’s motor neurons before entering the CNS (Katz et al., 2017).

Postexposure prophylaxis recommended by the World Health Organization (WHO) includes wound washing at the RABV exposure site, rabies immune globulin (RIG) administration, and rabies vaccine administration (World Health Organization, 2018). However, once the virus invades the CNS, rabies is nearly 100% fatal (Ugolini and Hemachudha, 2018).

Although facing a major challenge to cure patients from advanced rabies, researchers have made several attempts to treat symptomatic RABV (Smith et al., 2019). In 2004, a patient with rabies symptoms survived after applying a therapeutic approach called the Milwaukee protocol (Willoughby et al., 2005). Nevertheless, its effectiveness is controversial as no less than 31 failures have been reported using the same protocol (Zeiler and Jackson, 2016). Wang et al. (2011) investigate the direct injection of a recombinant RABV vaccine strain expressing GM-CSF (LBNSE-GMCSF) into the brains of mice 4–6days after they had been infected with a lethal dose of wild-type RABV. Studies by Chen et al. (2013) and Huang et al. (2015) demonstrated that in mice, intracranial injection of parainfluenza virus 5 (PIV5) expressing RABV glycoprotein elicited virus-neutralizing antibodies, enhanced the blood-brain barrier (BBB) permeability, and promoted the survivor ratio of mice infected with a lethal dose of RABV. A live RABV vaccine, on the other hand, is unlikely to be authorized for therapeutic use in human rabies patients. Most recently, de Melo et al. (2020) showed that a combination of RABV-neutralizing antibodies (RVNA) RVC20 and RVC58 could rescue symptomatic mice post RABV infection by concomitantly administration of RVNA in the CNS through intracerebroventricular infusion, sheding a light for an effective rabies therapy in humans.

Adeno-associated virus (AAV) has been used as a vector for gene therapy for many years (Flotte et al., 1993; Martinez-Navio et al., 2019). AAV could become the vector of choice for many gene therapy applications due to its simple structure, unique biology, and a lack of known disease associations (Naso et al., 2017; Rabinowitz et al., 2019). The number of clinical trials of AAV-based gene therapies initiated annually increased by more than four times from 2014 to 2017 (Kuzmin et al., 2021). Clinical data from more than 3,000 patients over more than 20years suggest that AAV-based gene therapy is safe and efficient. Recently, U.S. Food and Drug Administration approved two AAV-based therapies, namely Luxturna for a rare inherited retinal dystrophy and Zolgensma for spinal muscular atrophy (Kuzmin et al., 2021). The number of agents using AAV9 capsids for CNS delivery has increased since 2015, indicating the potential use of gene therapy for CNS diseases (Samaranch et al., 2019; Taghian et al., 2020).

In this study, we evaluate the efficiency of preexposure and postexposure prophylaxis of rabies with AAV expressing two RVNAs, CR57 and CR4098, in rodent models, both of which have presented a protective role during postexposure prophylaxis in a hamster model (Bakker et al., 2005; Marissen et al., 2005; Goudsmit et al., 2006). Our data show that delivery of AAV encoding RVNA CR57 can effectively reduce the mortality of RABV-infected rodents and may have applications for both preexposure and postexposure prophylaxis of rabies in future.

## Materials and Methods

### Viruses, Cells, Antibodies, Plasmids, and Animals

CVS-B2c is a laboratory-adapted RABV strain derived from the CVS-24 virus by passaging in BHK-21 cells (Wang et al., 2005). DRV-Mexico, isolated from a dog in Mexico, is a wild-type RABV strain (Dietzschold et al., 2000). BSR cells (a clone of BHK-21 cells) were maintained in DMEM containing 10% fetal bovine serum (FBS). Human embryonic kidney 293T cells (HEK-293T) were cultured in RPMI 1640 medium (Mediatech, Herndon, VA, United States) containing 10% FBS. RABV P protein-specific monoclonal antibody was prepared in our laboratory, as previously described (Sui et al., 2020). Fluorescein isothiocyanate (FITC)-conjugated antibodies against the RABV-N protein were purchased from Fujirebio (Malvern, PA). RABV CVS-B2c genomic backbone plasmid and helper plasmids were prepared in our laboratory, as previously described (Tian et al., 2016). The pAAV-CMV-eGFP and helper plasmids were obtained from Dr. Gang Cao, Huazhong Agricultural University, China (Liu et al., 2020). In addition, 6-week-old female BALB/c mice and Sprague-Dawley rats were purchased from the Center for Disease Control of Hubei Province, Wuhan, China. All animal experiments were approved by the Scientific Ethics Committee of Huazhong Agricultural University (permit number HZAURA-2019-009). The animal experiments involving the infection with RABV were carried out in the animal facility with ABSL-2 level at Huazhong Agricultural University.

### Construction of the Recombinant AAV Vectors

Three AAV9 plasmids were constructed, each containing a CMV promoter followed by a signal peptide and an anti-rabies immunoglobulin CR57 or CR4098 (Bakker et al., 2005; Goudsmit et al., 2006); EGFP replaced the immunoglobulin and served as a control. The resulting plasmids were referred to as AAV-CR57, AAV-CR4098, and AAV-EGFP. Recombinant AAVs were produced by transfection of HEK-293T cells with the selected AAV vector, helper1, and helper2 plasmids at a ratio of 1:1:1. Cells and culture supernatants were harvested at 36h post-transfection (hpt) and centrifuged for 5min at 1,000 *g*. The cell pellets were resuspended in RPMI 1640 and then subjected to four rounds of freeze/thaw in a dry ice bath and a 37°C water bath. The suspensions were centrifuged for 10min at 10,000 *g* to pellet the cell debris and then filtered through a 0.22-μm membrane. The AAV copy number was assessed by real-time PCR. Primers were designed based on the known sequence information. F: 5'-CGGCCTCAGTGAGCGA-3', R: 5'-GGAACCCCTAGTGATGGAGTT-3' (Meng et al., 2013).

### Confirmation of mAb Expression in Cells by Western Blotting

Human embryonic kidney 293T cells were infected with AAVs at a multiplicity of infection (MOI) of 5 and incubated at 37°C for 4days. Then, the cell culture medium was collected and enriched by protein A/G agarose beads (Smart Lifesciences, SM1505) and eluted with elution buffer (Sui et al., 2020). Next, the supernatants were collected, and a 1×SDS loading buffer was added. Then, samples were separated on 12% SDS polyacrylamide gels (SDS-PAGE) and transferred to PVDF membranes (Bio-Rad). Subsequently, the membranes were blocked with TBST supplemented with 5% (*w*/*v*) nonfat dry milk for 3h and then incubated with horseradish peroxidase (HRP)-conjugated rabbit anti-human IgG (Boster, Wuhan, China, BA1070) for 1h at 37°C. Finally, the blots were developed by a BeyoECL Star kit (Beyotime, P0018A). Images were taken by an Amersham Imager 600 (GE Healthcare) imaging system.

### Virus-Neutralizing Antibody Assay

Virus-neutralizing antibody titers were measured by using a fluorescent antibody virus neutralization (FAVN) assay as previously described (Luo et al., 2019). Briefly, quadruplicate samples of 3-fold serial dilutions of the test serum and the standard serum were prepared in 96-well microplates. The standard serum (2IU/ml) was obtained from Nancy Laboratory for Rabies and Wildlife in Malzéville, France. One hundred focus-forming units (FFU) of CVS-11 suspension were aliquoted into each well. The plates were incubated for 1h at 37°C, and then 2×10^4^ BSR cells were added to each well. The microplates were then incubated for 72h at 34°C. The cells were then fixed for 30min with 80% ice-cold acetone and stained with FITC-conjugated antibodies against the RABV N protein. The fluorescence was assessed under an IX51 fluorescence microscope (Olympus, Tokyo, Japan). The VNA titers were recorded as international units per milliliter using the standard curve from the reference serum.

### Mouse Immunization and Challenge Test

Six-week-old female BALB/c mice were randomly divided into three groups and immunized with 10^12^ vector genome AAVs *via* an intramuscular (i.m.) route. At the indicated time points post-immunization, serum was collected from the peripheral blood samples to quantify the VNA titers. The mice received a challenge infection with 30μl of 50×mouse 50% lethal doses (LD_50_) CVS-24 intracerebrally (i.c.) 24weeks after the primary immunization. Mice found to be moribund or lose more than 30% of their starting body weight were humanely euthanized.

### Construction and Rescue of the Recombinant RABV Expressing teLuc

The recombinant RABV expressing teLuc (RABV-teLuc) was constructed based on the RABV CVS-B2c strain as described previously (Zhang et al., 2013). Briefly, the luciferase teLuc coding sequence flanked by BsiW I and Nhe I restriction sites was inserted between the coding sequences of RABV G and L proteins to generate the plasmid pcDNA3.1-rB2c-teLuc. Notably, teLuc with a synthetic CTZ analog is more sensitive in rodent models than traditional luciferase (Yeh et al., 2017). For virus rescue, this plasmid in combination with helper plasmids expressing the N, P, G, and L proteins was transfected into mouse neuroblastoma cells using Lipofectamine™ 2000 (Invitrogen). The rescued recombinant RABV-teLuc virus was confirmed by direct fluorescence antibody assay (DFA) under an Olympus IX51 fluorescence microscope.

### Virus Titration

Viral titers were determined by DFA. Briefly, BSR cells were infected with 10-fold serial dilutions of the viruses in 96-well plates in quadruplicate. After incubation at 37°C for 48h, the cells were fixed with 80% acetone and then incubated with FITC-conjugated anti-RABV-N antibody for 1h at 37°C. Antigen-positive cells were visualized with an IX51 Olympus fluorescence microscope. Viral titers were calculated and are presented as the numbers of FFU/ml as previously described (Luo et al., 2019).

### Measurement of teLuc Luciferase Activity

Twenty-four mice were randomly divided into eight groups, with three mice in each group. Seven groups of mice were i.m. inoculated with 6×10^4^ FFU RABV-teLuc in the right hind limb, and one group of mice were i.m. injected with 100μl saline as a mock control. During the 3–9days post-infection (dpi), every one of the seven groups of infected mice was chosen every other day to collect their brains and spinal cords. The brains and spinal cords from the mock group were also collected at 9dpi and stored at −80°C. Brain and spinal cord samples from each mouse were separately placed in 2ml tubes containing 1ml DMEM to be homogenized at 4°C for 10min. Subsequently, the supernatants were individually collected and mixed with 0.01μmol diphenylterazine (DTZ; Med Chem Express) after centrifuging at 8,000*g* 4°C for 10min. The luciferase expressed by RABV-teLuc was detected using a GLOMAX 20/20 Luminometer (Promega). Supernatants from the infected cell cultures were mixed with 0.01μmol DTZ, and the luciferase activity was determined as described above.

### Bioluminescence Imaging of Animals Infected With RABV-teLuc

Twelve 6-week-old female BALB/c mice were randomly divided into four groups, with three mice in each group. Three groups of mice were i.m. inoculated with 50×mouse LD_50_ of RABV-teLuc, and the mock group was i.m. inoculated with the same volume of DMEM. One of the three groups of infected mice was taken every other day for intrathecal injection with 0.3μmol DTZ per mouse 3–5dpi. In addition, the mock group was inoculated with 0.3μmol DTZ per mouse. Five minutes after injection with DTZ, the mice in each group were imaged by an IVIS Lumina III (PerkinElmer).

Eighteen 6-week-old female rats were randomly divided into six groups, with three rats in each group. Five groups of rats were i.m. inoculated with 50×rat LD_50_ of RABV-teLuc. One group of rats was i.m. injected with 100μl saline as a mock control. During 3–7dpi, each of the five groups of RABV-infected rats was taken every other day for intrathecal injection with 0.3μmol DTZ per rat. The mock group was also inoculated with 0.3μmol DTZ per rat. Five minutes after injection with DTZ, the rats in each group were imaged by an IVIS Lumina III.

### Production and Purification of Rat RIG

Three rats were i.m. immunized with 10^7^ FFU of RABV vaccine strain LBNSE-dOG in the right hind legs (Pei et al., 2019). Four weeks later, the rats were sacrificed, and their blood was collected. The blood samples were placed at 37°C for 1h and then at 4°C for 12h before centrifuging at 3,000*g* for 10min. The serum was diluted 10-fold with 20mmol/L Na_3_PO_4_ and filtered with a 0.45-μm-pore-size filter. The filtered serum was then loaded onto a column of protein A&G agarose beads (Smart Lifesciences, SM1505) and eluted with elution buffer. The antibodies were concentrated using a centrifugal filter with a protein concentration tube with Molecular Weight Cut Off 50kD (Millipore) and then suspended in natural saline to preserve the antibody. VNA titers were measured by FAVN.

### AAV Administration in Animals

BALB/c mice (*n*=10/group) were i.m. inoculated with 50×mouse LD_50_ DRV-Mexico in the right hind limb. The mice were inoculated *via* i.c. with a dose of 40μl of 2.5×10^10^ vector genomes/μl (v.g./μl) of AAV-CR57 at 4, 5, and 6dpi, or intranasally (i.n.) with a dose of 20μl of 5×10^10^ v.g./μl of AAV-CR57 at 4dpi, or intravenously (i.v.) with a dose of 100μl of 1×10^10^ v.g./μl of AAV-CR57 at 4dpi. In addition, two groups of mice (*n*=10) were inoculated with AAV-CR4098 or AAV-EGFP at 4 dpi.

The SD rats (*n*=12/group) were i.m. inoculated with 50×rat LD_50_ DRV-Mexico in the right hind leg. At 4dpi, each group of rats received a single inoculation of 100μl of 4×10^10^ v.g./μl AAV-CR57, or 100μl of 4×10^10^ v.g./μl AAV-EGFP, or 100μl of 4×10^10^ v.g./μl AAV-CR57 plus 20IU/kg RIG or 100μl of 4×10^10^ v.g./μl AAV-EGFP plus 20IU/kg RIG. Briefly, after induction of anesthesia, the rat’s head was placed in a stereotactic frame and flexed in a prone position. The back of the neck was shaved, cleaned with 75% alcohol, and then injected with the indicated solutions into the cisterna magna as described previously (Samaranch et al., 2012).

### Western Blotting

Brains were lysed in RIPA buffer (Beyotime, P0013D) mixed with 1× protease inhibitor cocktail (Roche). Brain lysates were separated on 12% SDS-PAGE gels and then transferred to PVDF membranes (Bio-Rad). The membranes were blocked with TBST supplemented with 5% (*w*/*v*) nonfat dry milk for 3h and then incubated with primary antibodies, including anti-RABV N protein (laboratory prepared, 1:5,000) or anti-GAPDH (ProteinTech, 60004-1-Ig, 1:5,000), overnight at 4°C. After rinsing, the membranes were incubated with HRP-conjugated goat anti-mouse IgG (Boster, Wuhan, China, BA1051) for 1h at 37°C and then developed by a BeyoECL Star kit (Beyotime, P0018A). Images were taken by an Amersham Imager 600 (GE Healthcare) imaging system.

### Immunohistochemistry Analysis

Brain specimens were fixed in 4% paraformaldehyde for 48h at 4°C and then dehydrated in PBS supplemented with 30% sucrose for 48h at 4°C. The tissue sections were embedded in paraffin wax and mounted on slides, and the slides were heated to 60°C, deparaffinized, and rehydrated in xylene and graded ethanol, heated for antigen retrieval, and treated with an endogenous peroxidase blocking solution. The sections were then incubated with primary anti-RABV P antibody (laboratory prepared, 1:500) overnight at 4°C. Next, the sections were incubated with HRP-conjugated antirabbit secondary antibodies (BioPM, PMK-013-090, 1:200) for 30min, followed by two washes in PBS. After washing, the sections were incubated with diaminobenzidine (Servicebio, G1211) for color development and then photographed and analyzed using an XSP-C204 microscope (CIC).

### Statistical Analysis

GraphPad Prism 8 (GraphPad Software, Inc., CA, United States) was used for data analysis. Unpaired two-tailed Student’s *t*-test was used to analyze the statistical significance. Kaplan-Meier survival curves were used to determine the statistical significance of the survivor ratio in the challenge test. Statistically significant differences are represented as ^*^p<0.05, ^**^p<0.01, ^***^p<0.001, and ^****^p<0.0001; ns means no significant difference.

## Results

### Characterization of Recombinant AAVs Expressing Anti-RABV Antibodies

**Figure 1:**
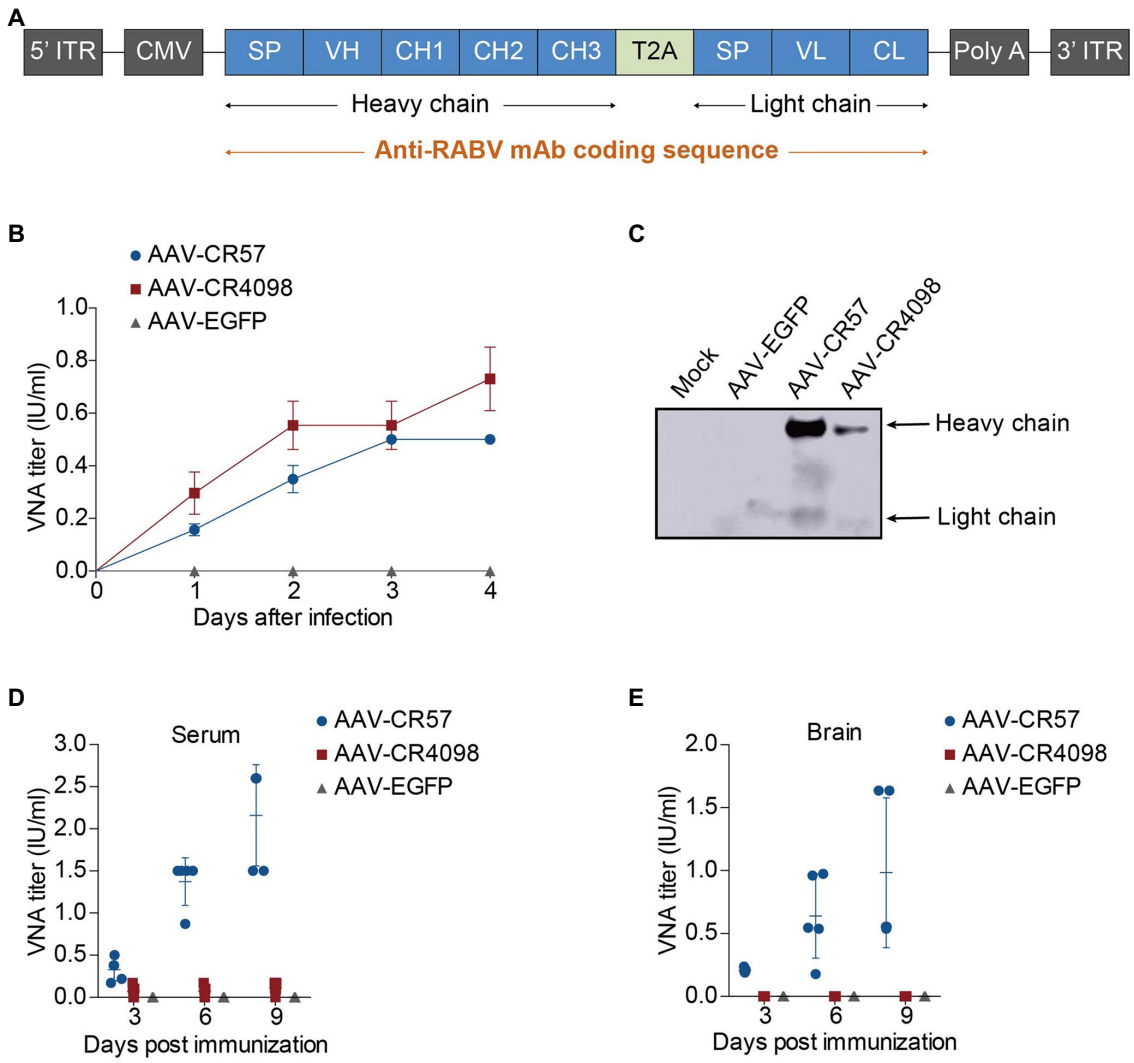
Construction and characterization of recombinant adeno-associated viruses (AAVs) expressing rabies virus-neutralizing antibodies (RVNA). **(A)** A schematic diagram describing the construction of the AAV-RVNA vector. The AAV expression vector contains a CMV enhancer/promoter, a signal peptide (SP), and an anti-rabies virus (RABV) monoclonal antibody (CR57 or CR4098) sequence. **(B)** One-step growth curves for AAVs on HEK-293T cells at a multiplicity of infection (MOI) of 5 are shown. **(C)** Western blotting analysis of RVNA expression in HEK-293T cells infected with AAVs. **(D)** Groups of 6-week-old female BALB/c mice (*n*=5) were i.c. inoculated with 10^12^ vector genome of AAVs. At the indicated time points post-inoculation, the serum **(D)** and brain **(E)** were collected, and the VNA titers in these samples were determined by using the fluorescent antibody virus neutralization (FAVN) test as described in the Materials and Methods.

To determine whether AAV-expressing antibodies can be used for preexposure and postexposure prophylaxis of rabies, the encoding sequences of two well-known RABV-specific virus-neutralizing antibodies, CR57 and CR4098 (Bakker et al., 2005; Marissen et al., 2005), as well as EGFP (as a control), were cloned into the AAV9 vector. A schematic description of the construction of different recombinant AAVs (AAV-CR57, AAV-CR4098, and AAV-EGFP) is shown in Figure 1A. HEK-293T cells were infected with different AAVs at an MOI of 5, and VNA titers in the culture supernatants were monitored over 4dpi. At 3dpi, CR4098 and CR57 titers in the culture supernatants were equal to or greater than 0.5 international units/ml (IU/ml), which is the minimum protection level against RABV infection defined by the WHO (Figure 1B). The culture supernatants were collected and enriched at 4dpi. Expression of the heavy chain and light chain was detected by Western blotting with HRP-conjugated rabbit anti-human IgG (Boster, Wuhan, China, BA1070). As shown in Figure 1C, the mAb expression of AAV-CR57 was much higher than that of AAV-CR4098 in HEK-293T cells. Therefore, we further evaluated the VNA levels generated by the AAVs *in vivo*. The AAVs were inoculated intracerebrally (i.c.) into BALB/c mice; at each time point (3, 6, and 9 dpi), mice were humanely sacrificed (*n*=5/group), and the VNA levels in sera (Figure 1D) and brains (Figure 1E) were measured. The results showed that AAV-CR57 generated a protective level of VNA in mouse serum and brains post i.c. inoculation. However, mAb produced by AAV-CR4098 in the serum and the brain was almost undetectable.

### Intramuscular Immunization With AAV-CR57 Results in Long-Lasting Antibody Production in Mice

**Figure 2:**
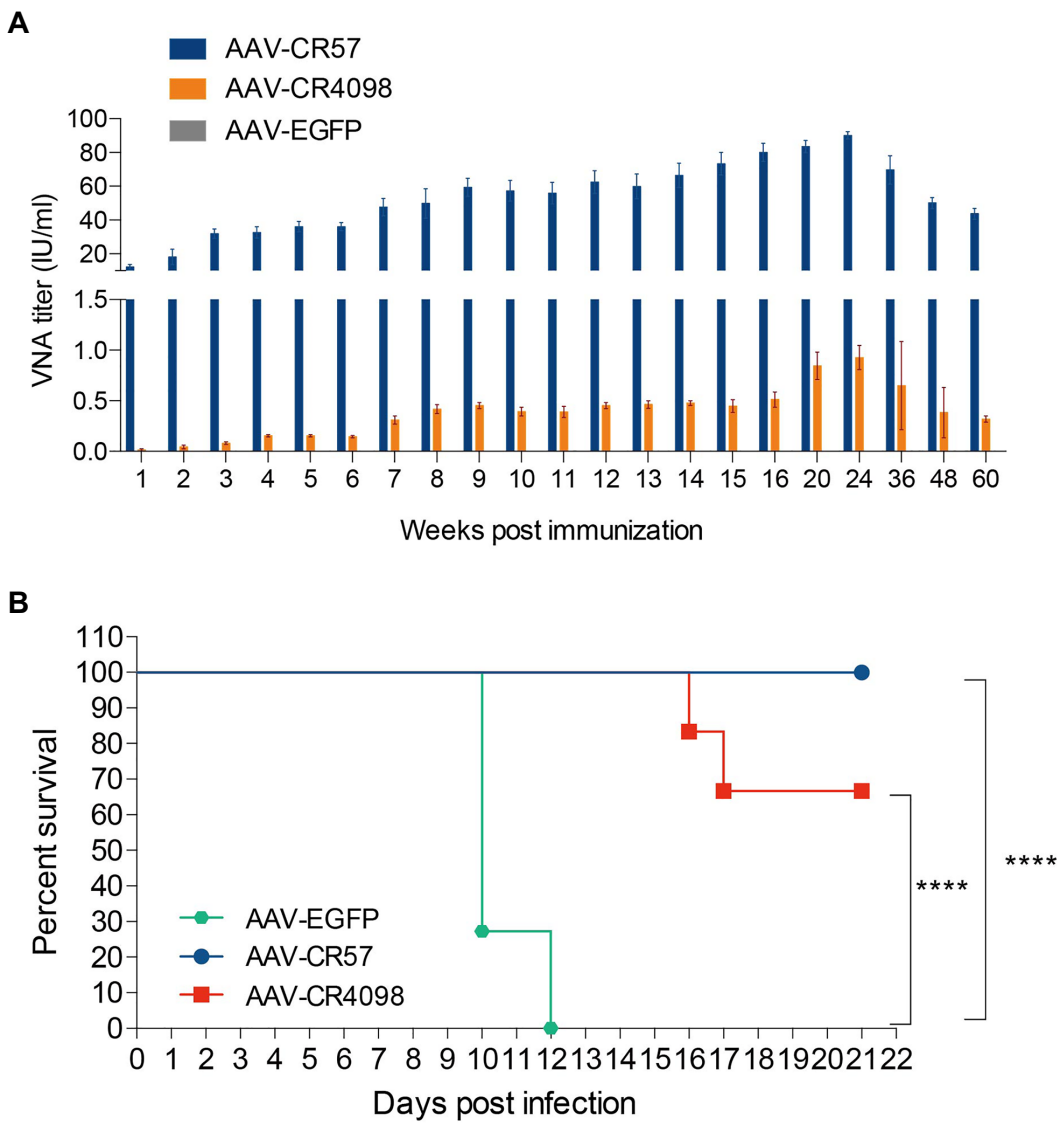
Preexposure prophylaxis of rabies by using AAVs expressing RVNA. **(A)** RVNA levels in serum following the administration of AAV expressing CR57, CR4098, or EGFP. Groups of 6-week-old female BALB/c mice (*n*=10) were i.m. immunized with a 10^12^ vector genome of AAV. The serum was collected from the peripheral blood samples at the indicated time points post-immunization, and the VNA titers were quantified by the FAVN test. **(B)** At 24weeks post-primary inoculation, the mice (*n*=12) were i.c. challenged with 50×mouse LD_50_ CVS-24 and were then observed daily for 3weeks. The number of survivors in each group was recorded. ^****^p < 0.0001.

Since AAV-CR57 and AAV-CR4098 can generate mAbs *in vivo* and *in vitro*, the effect of the AAVs on antibody production *via* i.m. inoculation was evaluated in a mouse model. Three groups of BALB/c mice (*n*=12/group) were immunized, i.m. with 10^12^ vector genomes of AAV-CR57, AAV-CR4098, or AAV-EGFP. At indicated time points post-immunization, blood samples were collected, and VNA titers were measured using the FAVN test. The mice immunized with AAV-CR57 maintained significantly higher VNA titers than the AAV-CR4098-immunized mice at all the time points up to 60weeks post-infection (wpi). The antibody levels in the mice reached a peak (92IU/ml) at 24 wpi (Figure 2A).

Furthermore, the protective effect of AAVs against virulent RABV challenge was evaluated. At 24 wpi, all of the mice (*n*=12) were i.c. challenged with 50×mouse LD_50_ of CVS-24 and then monitored for another 3weeks (Figure 2B). All of the mice in the AAV-EGFP group succumbed to rabies within 12days, while 100% of the mice immunized with AAV-CR57 were protected from the lethal challenge compared with only 66% of the AAV-CR4098 group. These results demonstrated that preexposure immunization with AAV-CR57 could result in sustained antibody production and provide long-term protection.

### *In vitro* Characterization of RABV-teLuc and Trace the Migration Dynamic of RABV With RABV-teLuc in a Mouse Model

**Figure 3:**
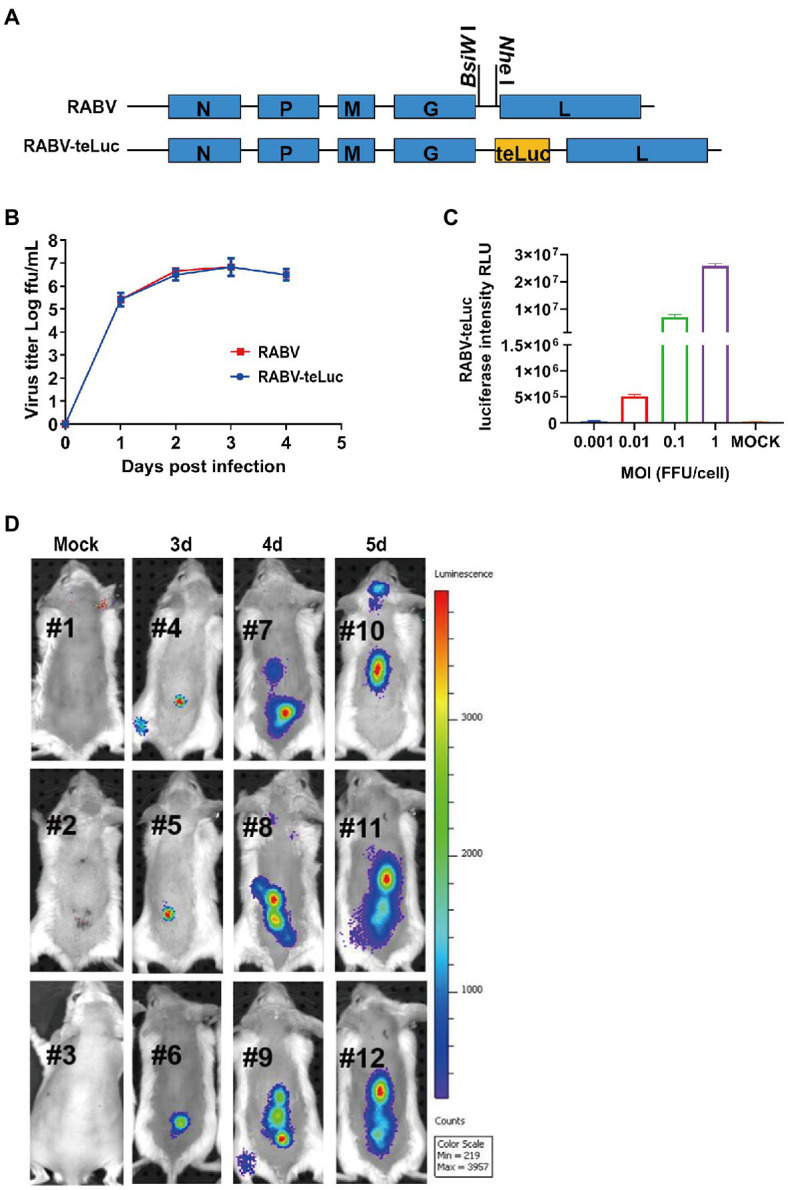
The migration dynamics of RABV in mice post i.m. inoculation with a recombinant RABV expressing teLuc. **(A)** Schematic diagram showing the construction of recombinant RABV expressing teLuc, termed RABV-teLuc. **(B)** Single-step growth curves of RABV-teLuc and the parent virus in BSR cells infected at an MOI of 0.01. **(C)** Expression of teLuc in BSR cells infected with the indicated dose of RABV-teLuc at 24h post-infection (hpi). **(D)** Early detection of RABV-teLuc infection in a mouse model. Twelve 6-week-old BALB/c mice were randomly divided into four groups, with three mice in each group. Three groups of mice were i.m. inoculated with 50×mouse LD_50_ RABV-teLuc, and the mock group was i.m. inoculated with the same volume of DMEM. During 3–5 dpi, one of the three groups of RABV-infected mice was taken every other day for intrathecal injection with 0.3μmol diphenylterazine (DTZ) per mouse. The mock group was inoculated with 0.3μmol DTZ per mouse. The mice in each group were imaged by an IVIS Lumina III 5min post-injection with DTZ.

To track the migration dynamic of RABV in mice, a recombinant RABV encoding teLuc was constructed. As shown in Figure 3A, the coding sequence of teLuc was inserted into the genome of RABV CVS-B2c strain between the G and L genes, resulted in RABV-teLuc. The growth curves of RABV-teLuc in BSR cells demonstrated that RABV-teLuc replicated as efficiently as the parent virus CVS-B2c (Figure 3B) and that the expression of luciferase in RABV-teLuc-infected BSR cells (MOI of 0.001, 0.01, 0.1, and 1) at 24h post-infection (hpi) was dose-dependent (Figure 3C). Post i.m. infection with RABV-teLuc in the mouse hind legs, the migration dynamic in the mouse bodies was monitored by a bioluminescence imaging system (Figure 3D). The results showed that RABV-teLuc was detected as early as 3 dpi in the spinal cord of infected mice. From 3 to 5 dpi, RABV migrated from the spinal cords to the brain. At 5 dpi, RABV in one of the mice (*n*=3) was found to invade the brain. These data were consistent with other detection methods like RT-qPCR and RPA-CRISPR (Ren et al., 2021a) and provided the basis for choosing the appropriate time point to deliver AAV-CR57 into the RABV-infected mice.

### Protection of AAV-CR57 by Different Administration Ways in a Mouse Model

**Figure 4:**
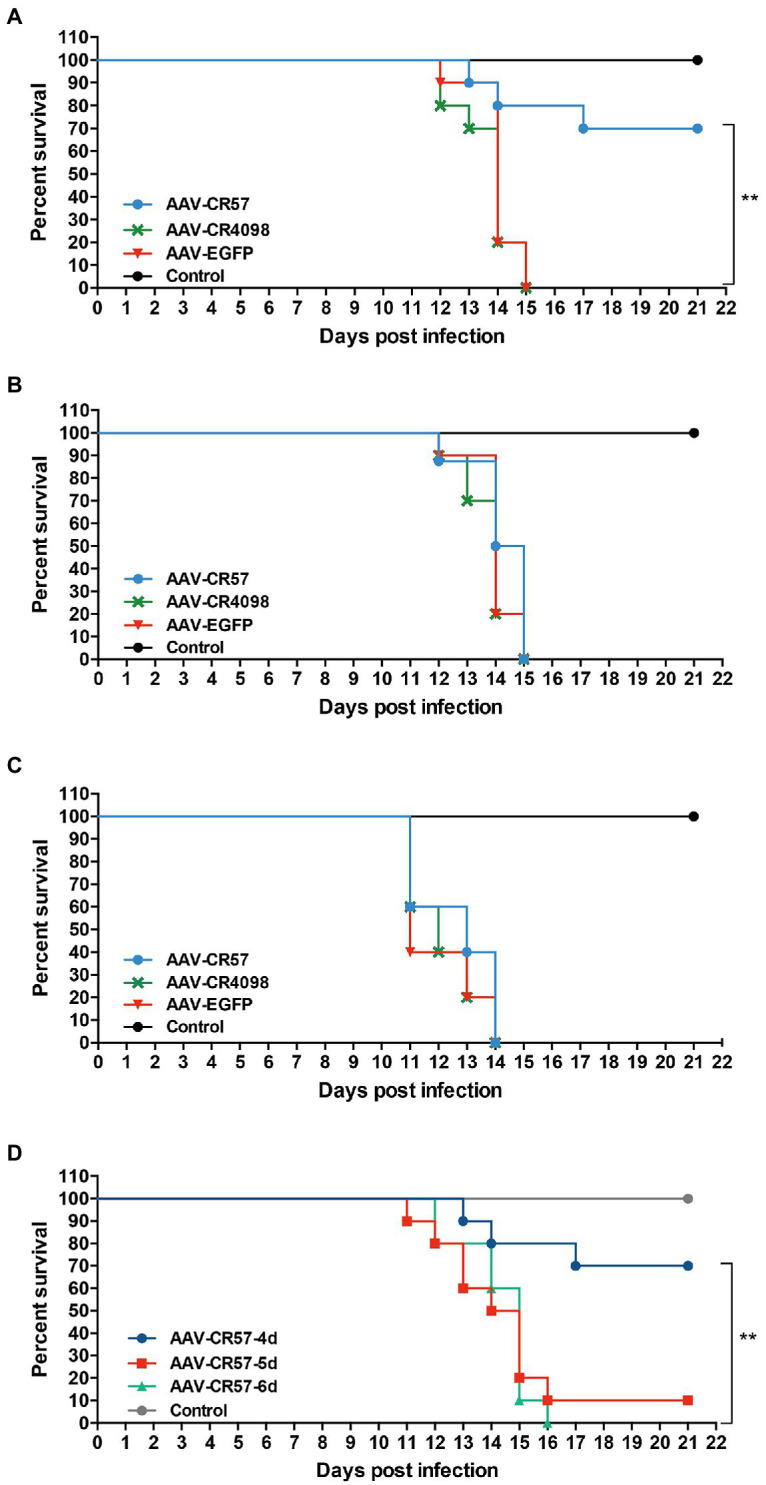
Postexposure prophylaxis for rabies with AAV-expressing RVNA in a mouse model. Groups of 6-week-old female BALB/c mice (*n*=10) were i.m. injected with 50×mouse LD_50_ DRV. At 4 dpi, the mice was inoculated with 10^12^ vector genomes of different AAVs *via* i.c. **(A)**, i.v. **(B)** or i.n. route **(C)**. **(D)** Groups of 6-week-old female BALB/c mice (*n*=10) were i.m. injected with 50×mouse LD_50_ DRV and then i.c. inoculated with 10^12^ vector genomes AAV-CR57 at 4, 5, and 6days post-inoculation. The mock group was inoculated with the same volume of DMEM. Mice were monitored once daily for 3weeks. The number of survivors in each group was recorded. Kaplan-Meier survival curves were used to determine the statistical significance of the survivor ratio. ^**^p < 0.01.

To evaluate the therapeutic efficacy of AAV-CR57 in a rabies postexposure prophylaxis mouse model, mice were injected, i.m. with 50×mouse LD_50_ DRV-Mexico, and then treated with AAV-CR57, AAV-CR4098, or AAV-EGFP (*via* i.c., i.v., or i.n.) at 4 dpi (*n*=10). The results showed that intracerebral administration with AAV-CR57 at 4 dpi significantly improved the 3-week survival ratio of the infected mice (70%) over those treated with AAV-EGFP (0%; Figure 4A). In contrast, administration with AAV-CR57 by i.v. or i.n. route did not affect mouse survival (Figures 4B,C). However, inoculation with AAV-CR57 by i.c. at 5 and 6 dpi did not enhance the survival ratio of the infected mice over the negative control (Figure 4D). These data demonstrated that direct delivery of AAVs to the CNS no later than 4 dpi is necessary for a therapeutic effect in RABV-infected mice.

**Figure 5:**
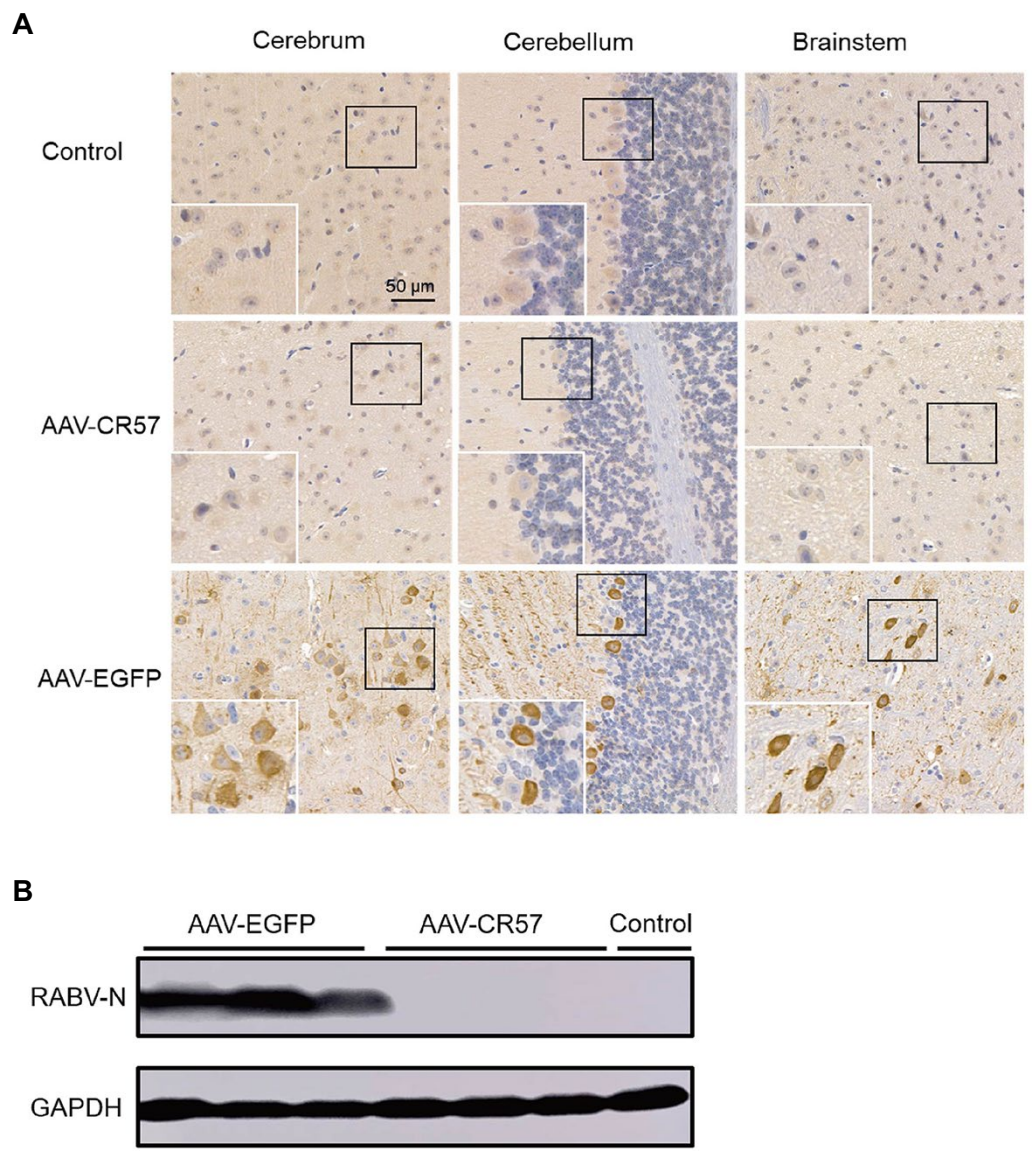
Viral load in mouse brains post i.c. inoculation with AAVs expressing CR57 or EGFP. Groups of 6-week-old female BALB/c mice (*n*=10) were i.m. injected with 50×mouse LD_50_ DRV and i.c. inoculated with 10^12^ vector genome of AAVs at the 4 dpi. The mock group was inoculated with the same volume of DMEM at 4 dpi. At 12 dpi, the brains were collected, and half of the brain was fixed, paraffin-embedded, sectioned, and analyzed by immunohistochemistry by staining with antibodies against RABV-P **(A)**. Scare bar, 50μm. The other half of the brain was analyzed for RABV-N levels by Western blotting **(B)**.

Moreover, mouse brains were collected for viral quantification and immunohistochemical (IHC) analysis at 12 dpi (*n*=3). IHC analysis showed no RABV-P-positive cells were observed in the cerebrum, cerebellum, or brainstem of the mice in the AAV-CR57 group. In contrast, significantly more RABV-P-positive cells were observed in the AAV-EGFP group (Figure 5A). The viral protein in the mouse brains was further determined by Western blotting. As shown in Figure 5B, no RABV N protein was detected in the brains of mice treated with AAV-CR57, while RABV N protein was observed in the brains of mice treated with AAV-EGFP. Collectively, these results indicated that AAV-CR57 could protect mice from RABV infection.

### Protection of AAV-CR57 by the Intrathecal Route in a Rat Model

**Figure 6:**
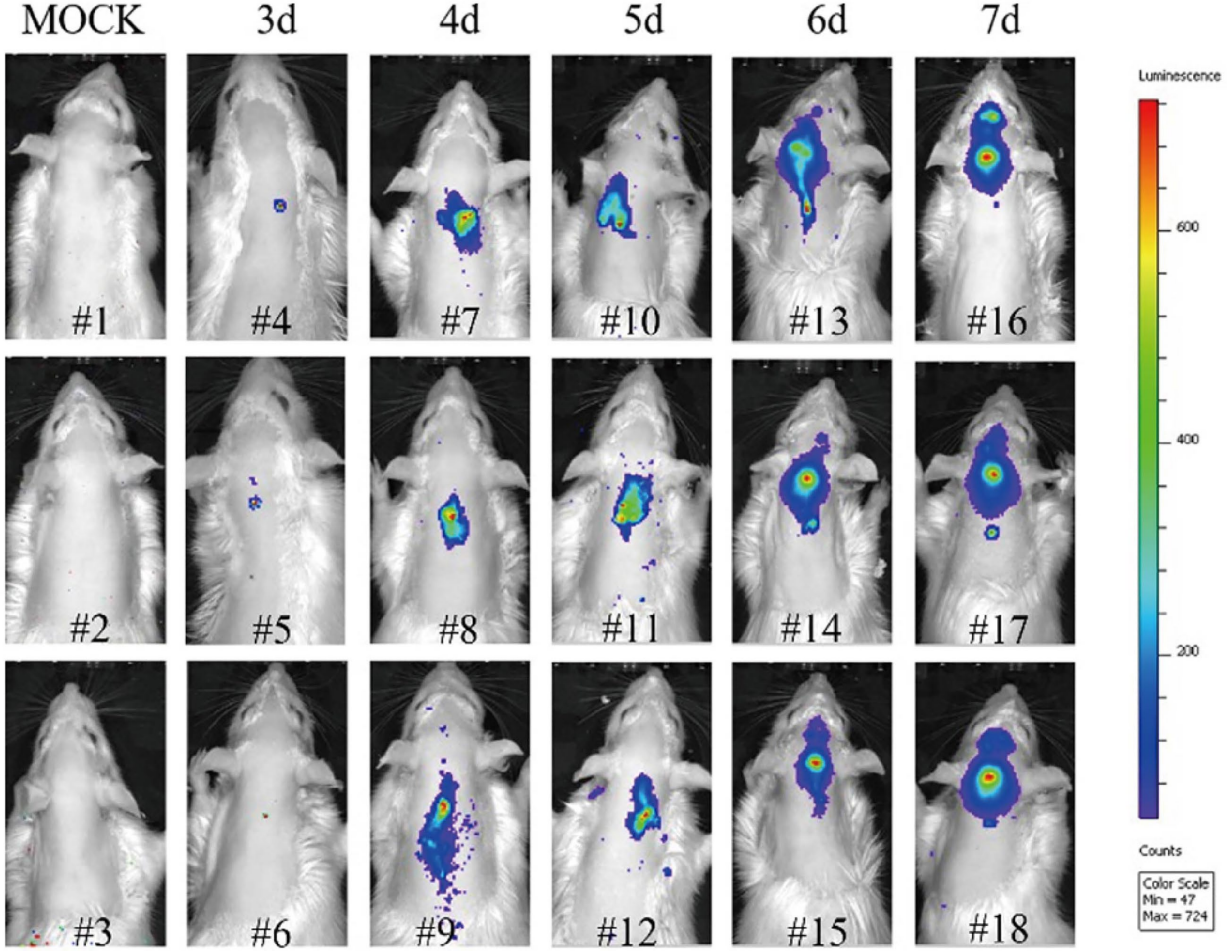
The migration dynamics of RABV post i.m. infection in a rat model. Eighteen 6-week-old Sprague-Dawley (SD) rats were randomly divided into six groups, with three rats in each group. Five groups of rats were i.m. inoculated with 50×rat LD_50_ RABV-teLuc, and the mock group was i.m. inoculated with the same volume of DMEM. During 3–7 dpi, one of the five groups of infected rats was taken every other day for intrathecal injection with 0.3μmol DTZ per rat. The mock group was inoculated with 0.3μmol DTZ per rat. Five minutes after injection with DTZ, the rats in each group were imaged by an IVIS Lumina III.

According to the above results, the i.c. route was the most effective administration way of AAV-CR57 for rabies postexposure prophylaxis in the mouse model. However, it is not quite feasible to deliver AAV-CR57 to the rabies patient by i.c. route. Alternatively, the intrathecal administration is commonly used in rabies treatment for humans (Basgoz and Frosch, 1998; Appolinario and Jackson, 2015). However, it is not easy to do the intrathecal injection in mice due to its tiny body size, and the rat model was therefore chosen to simulate the rabies treatment in humans by intrathecal administration in our study. Similarly, to trace RABV spread in rats post i.m. infection, bioluminescence imaging was performed in RABV-teLuc-infected rats. As shown in Figure 6, RABV-teLuc was detected as early as 3 dpi in the spinal cord of infected rats (*n*=3), and the viruses almost reached the brains of infected rats (*n*=3) at 5 dpi and invaded into the brains of infected rats (*n*=3) at 6 and 7 dpi. Bioluminescence images of these rats indicated that 4 dpi could be an appropriate injection window for rabies treatment in a rat model.

**Figure 7:**
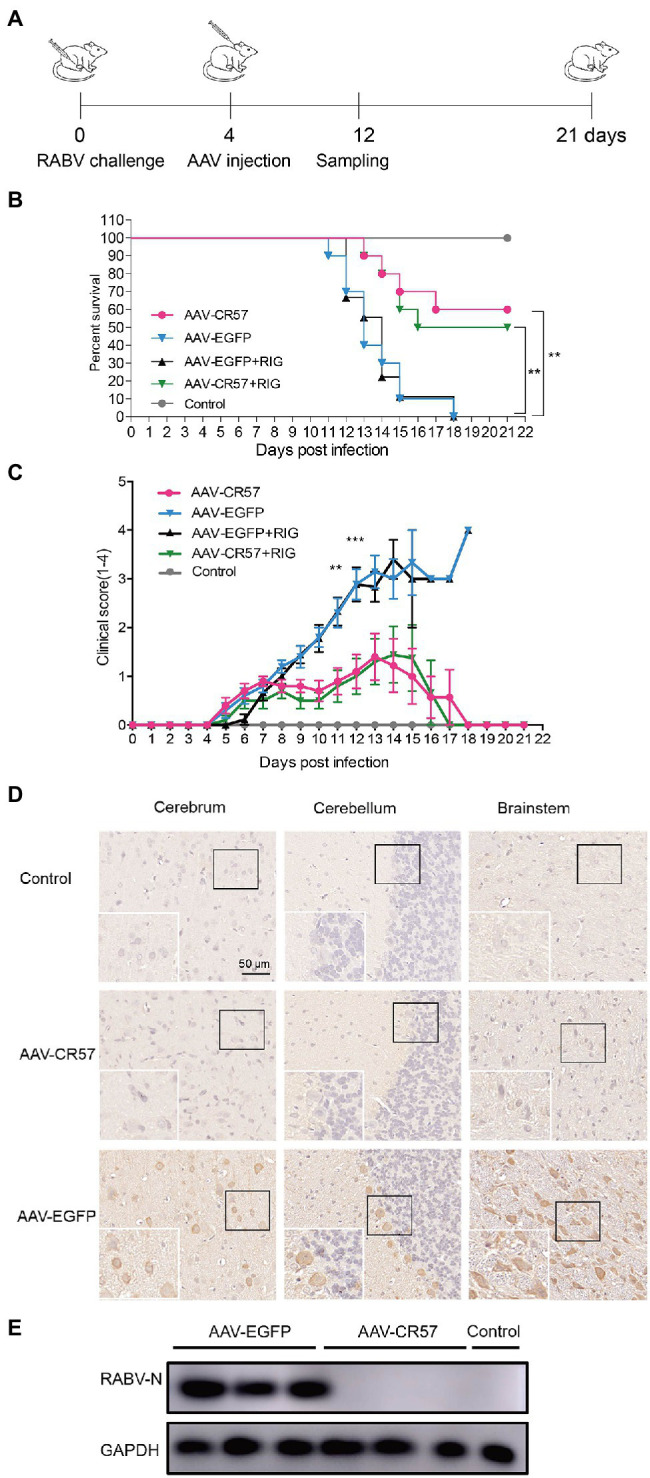
Postexposure prophylaxis with AAV-CR57 for DRV-infected rats. **(A)** Flow chart of the therapeutic experiment of AAV-CR57 in a rat model. Groups of 6-week-old female SD rats (*n*=10) were i.m. inoculated with 50×rat LD_50_ DRV and intrathecally immunized with 4×10^12^ vector genome of AAVs or together with 20IU/kg rabies immune globulin (RIG) at the indicated time points post-inoculation. The mock group was i.m. inoculated with the same volume of DMEM and intrathecally immunized with the same volume of DMEM at the indicated time points post-inoculation. Survival ratio **(B)** and clinical score **(C)** were monitored once daily for 3weeks (0: no apparent changes; 1: weight loss; 2: hind limb ataxia; 3: hind limb paralysis; and 4: paralysis). At 12 dpi, the brains were collected. Half of the brain was fixed, paraffin-embedded, sectioned, and analyzed by immunohistochemistry with antibodies against RABV-P **(D)**. The other half of the brain was homogenized and analyzed by Western blotting **(E)**. Detailed information about RIG was described in Production and Purification of Rat RIG section. ^**^p < 0.01; ^***^p < 0.001.

The RIG was widely used along with the rabies vaccine for rabies postexposure prophylaxis in humans, which provides rapid antibodies until the body can respond to the vaccines. Therefore, the strategy of delivering AAV-CR57 alone, AAV-CR57+rat RIG, AAV-EGFP+rat RIG, or AAV-EGFP alone to the CNS *via* intrathecal inoculation was assessed in a rat model, and the rat RIG was prepared and purified as described in the Materials and Methods section. Four groups of SD rats (12 rats/group) were inoculated, i.m. with 50×rat LD_50_ DRV-Mexico. At 4 dpi, they were treated with AAV-CR57, AAV-EGFP, AAV-CR57+RIG (20IU/kg), or AAV-EGFP+RIG (20IU/kg) and then monitored daily for 3weeks, and the flow chart of experimental design was as shown in Figure 7A. The survival rate was 60% in rats treated with AAV-CR57 and 50% in those treated with AAV-CR57+RIG. However, all rats treated with AAV-EGFP or AAV-EGFP+RIG succumbed to rabies by 18 dpi (Figure 7B). Moreover, the rabies symptoms (including weight loss, hind limb ataxia, hind limb paralysis, and paralysis) of the AAV-EGFP or AAV-EGFP+RIG inoculated rat appeared at 5 dpi and became exacerbated until death at 18 dpi, while five of the rats inoculated with AAV-CR57 or AAV-CR57+RIG displayed mild symptoms which occurred at 6 dpi and recovered from 9 to 13 dpi (Figure 7C). These data indicated that intrathecal inoculation with RIG alone had no obvious therapeutic effect in the RABV infected rats at 4 dpi, nor did it have any therapeutic benefit over AAV-CR57 administration alone.

Furthermore, the rat brains were collected for viral quantification and IHC analysis at 12 dpi (*n*=3). IHC analysis showed that no RABV-P-positive cells were observed in the cerebrum, cerebellum, or brainstem of rats in the AAV-CR57 group. In contrast, significantly more RABV-P-positive cells were observed in the AAV-EGFP group (Figure 7D). The viral protein in the rat brains was further determined by Western blotting, and no RABV N protein was detected in the brains of rats treated with AAV-CR57, while RABV N protein was detected in the brains of rats treated with AAV-EGFP (Figure 7E). Collectively, these results indicated that AAV-CR57 could increase the survivor ratio through intrathecal inoculation.

## Discussion

Several limitations still exist for the timely clearance of RABV in the CNS. Early detection of RABV using traditional methods remains difficult as the virus replicates relatively slowly before invading the brain. Delivery of VNAs to the CNS, due to the protection of the BBB, presents another obstacle for timely neutralization of RABV. This study investigated the efficacy of AAVs expressing anti-RABV antibodies for preexposure and postexposure prophylaxis of rabies in rodent models.

Mild clinical symptoms of rabies such as weight loss could begin to appear at 6 or 7 dpi in RABV-infected mice (Tian et al., 2017). However, RABV had already entered into the brain by that time (Tian et al., 2017), and the initiation of delivery of AAV-expressing RVNA may have been too late. Early diagnosis allows for the timely initiation of appropriate delivery and increases the chances of recovery. Bioluminescence imaging showed a positive viral signal in the brains of infected rats from 6 to 7 dpi, whereas the viral signal was observed in the spinal cords of infected rats at 4 dpi (Figure 6). Combining the current methods with advanced detection techniques, such as RPA-CRISPR (Ren et al., 2021a), could improve therapeutic efficiency.

As indicated by the imaging results, RABV was presented in the spinal cords of the infected rats by 4 dpi and had invaded the brain at 6 dpi (Figure 6). Thus, there is a short window (approximately 2days) during which time treatment can be initiated to interfere with RABV progression into the brain. According to our results, 70% survival rate was observed in RABV-infected mice that were intracerebrally inoculated with AAV-CR57 at 4 dpi. However, administration with AAV-CR57 at 5 and 6 dpi did not enhance the survival rate (Figure 4D) because it usually took 2–3days to express enough VNA to neutralize RABV after intrathecal delivery of AAV-CR57 (Figures 1D,E). In this situation, the expression of VNA in the mouse brain could not keep up with the speed of viral transmission, causing a reduction in the survival ratio. Thus, the delivery of AAV expressing VNA to the CNS no later than 4 dpi is necessary for a therapeutic effect in RABV-infected mice. Notably, AAV-CR57 did not affect survival by i.v. or i.n. administration (Figures 4B,C). Most likely, only a small proportion of AAV-CR57 could pass through the BBB post i.v. or i.n. administration due to the protection of BBB. Therefore, intracerebral inoculation is the best strategy for AAV-CR57 dilvery in a mouse model.

It is not practical to inoculate AAV-CR57 into a human *via* intracerebral route. Thus, we evaluate the therapeutic effect of AAV-CR57 in a rat model *via* intrathecal inoculation. To supply VNA during the incubation period after inoculation with AAV-CR57, we injected AAV-CR57 together with rat-derived RIG into the CSF *via* intrathecal inoculation. Notably, a dose of RIG at 20IU/kg body weight is typically recommended by the WHO for postexposure prophylaxis of rabies, and RIG is administered at the site of the bite(s) (World Health Organization, 2018). Unfortunately, our results suggested that there was no obvious difference between AAV-CR57 and AAV-CR57+RIG administration. According to a previous study, IgG was eliminated from the rat CSF by bulk flow at a half-life of approximately 47min and a clearance of approximately 29ml/day/kg. The eliminated IgG was transferred from the CSF into the blood circulation within 24h after ICV dosing (Noguchi et al., 2017). We speculate that the short half-life of RIG in CSF might impede its neutralization effect. Before the VNA expression of AAV-CR57, the virus in the spinal cord was not effectively neutralized because the RIG did not remain in the CSF long enough after a single-dose injection.

The gene sequences of two well-known RABV-specific neutralizing antibodies, CR57, and CR4098, were cloned into AAVs and tested *in vitro* and *in vivo*. These two antibodies have been well-studied in animal models and have shown potential as an alternative to RIG in postexposure prophylaxis of rabies (Franka et al., 2017). However, we found that 8weeks after i.m. administration of the 10^12^ vector genome, the VNA titer of AAV-CR4098-immunized mice was only 0.5IU/ml compared with 40IU/ml for the AAV-CR57-immunized mice (Figure 2A). Only AAV-CR57 generated a protective level of VNA in the serum and brain tissue after i.c. administration of the 10^12^ AAV vector into mouse brains (Figure 4A). It is not currently understood why AAV-CR4098 generates such low VNA titers compared with AAV-CR57. Newly developed mAbs such as RVC20 and RVC58 were found to have a higher potency than CR57 and CR4098 (De Benedictis et al., 2016; de Melo et al., 2020; Hellert et al., 2020). Combined utilization of these mAbs may improve the effectiveness of postexposure prophylaxis.

Due to the complicated structure of the CNS, timely clearance of the most neurotropic viruses in the CNS is still a major challenge. Researchers have attempted to develop antiviral drugs and antibodies, and even new materials against neurotropic viruses (Lofano et al., 2018; Ren et al., 2021b). However, some defects limit the efficacy of these agents, especially the obstruction of the BBB. This study demonstrated that AAV-CR57 is a practical strategy for preexposure and postexposure prophylaxis of rabies in the rodent model. Thus, this strategy can potentially be applicable for other infectious diseases caused by neurotropic pathogens.

## Data Availability Statement

The original contributions presented in the study are included in the article/supplementary material, further inquiries can be directed to the corresponding authors.

## Ethics Statement

The animal study was reviewed and approved by the Scientific Ethics Committee of Huazhong Agricultural University.

## Author Contributions

FH and LZ: conceptualization, methodology, validation, and visualization. FH: formal analysis. FH, MR, JP, HM, BS, QW, and BC: investigation. LZ: resources and supervision. FH and MR: data curation. FH and RY: writing – original draft preparation. FH, MZ, and LZ: writing – review and editing. HZ and LZ: project administration. ZF and LZ: funding acquisition. All authors contributed to the article and approved the submitted version.

## Conflict of Interest

The authors declare that the research was conducted in the absence of any commercial or financial relationships that could be construed as a potential conflict of interest.

## Publisher’s Note

All claims expressed in this article are solely those of the authors and do not necessarily represent those of their affiliated organizations, or those of the publisher, the editors and the reviewers. Any product that may be evaluated in this article, or claim that may be made by its manufacturer, is not guaranteed or endorsed by the publisher.
